# Investigating implementation of school health policies through a health equity lens: A measures development study protocol

**DOI:** 10.3389/fpubh.2022.984130

**Published:** 2022-11-30

**Authors:** Gabriella M. McLoughlin, Callie Walsh-Bailey, Chelsea R. Singleton, Lindsey Turner

**Affiliations:** ^1^College of Public Health, Temple University, Philadelphia, PA, United States; ^2^Implementation Science Center for Cancer Control and Prevention Research Center, Brown School, Washington University in St. Louis, St. Louis, MO, United States; ^3^School of Public Health and Tropical Medicine, Tulane University, New Orleans, LA, United States; ^4^College of Education, Boise State University, Boise, ID, United States

**Keywords:** health equity, measurement, protocol, nutrition, school wellness, health policy, policy implementation, implementation science

## Abstract

**Background:**

School-based policies that ensure provision of nutrition, physical activity, and other health-promoting resources and opportunities are essential in mitigating health disparities among underserved populations. Measuring the implementation of such policies is imperative to bridge the gap between policy and practice. Unfortunately, limited practical, psychometrically strong measures of school policy implementation exist. Few available explicitly focus on the issues of equity and social justice as a key component of implementation, which may result in underassessment of the equity implications of policy implementation. The purpose of this study is to develop equity-focused measures in collaboration with practitioners, researchers, and other key implementation partners that will facilitate evaluation of policy implementation determinants (i.e., barriers and facilitators), processes, and outcomes.

**Methods:**

We will actively seek engagement from practitioners, researchers, and advocacy partners (i.e., stakeholders) who have expertise in school health policy throughout each phase of this project. We propose a multi-phase, 1-year project comprising the following steps: (1) selection of relevant constructs from guiding frameworks related to health equity and implementation science; (2) initial measure development, including expert feedback on draft items; (3) pilot cognitive testing with representatives from key target populations (i.e., school administrators, teachers, food service staff, and students and parents/guardians); and (4) measure refinement based on testing and assessment of pragmatic properties. These steps will allow us to establish initial face and content validity of a set of instruments that can undergo psychometric testing in future studies to assess their reliability and validity.

**Discussion:**

Completion of this project will result in several school policy implementation measurement tools which can be readily used by practitioners and researchers to evaluate policy implementation through a health equity lens. This will provide opportunities for better assessment and accountability of policies that aim to advance health equity among school-aged children and their families.

**Trial registration:**

Open Science Framework Registration doi: 10.17605/OSF.IO/736ZU.

## Introduction

Policy, systems, and environmental interventions represent a key opportunity for advancing population health due to their broad reach across societal layers. Given that health conditions in childhood frequently persist into adulthood, establishing structures and systems that facilitate opportunities for health-promoting behavior such as healthy eating and physical activity is a critical step to mitigating risk for chronic disease (1–3). Further, given the disproportionate risk of overweight and obesity among children from underserved populations in the United States, such as non-white racial/ethnic groups and those living in poverty (4, 5), policy interventions may be the optimal vehicle for change. Several policies at the school and district level, such as the United States Child Nutrition and Women, Infants, and Children (WIC). Reauthorization Act, United States Department of Agriculture final rule and Universal School Meals (USM) under the Community Eligibility Provision have been introduced within the last two decades mandating standards for nutrition and wellness policy in schools and districts serving low-income student populations (6–8). Specifically, USM allow schools to provide free breakfast and lunch to all students within high poverty schools (where >40% of students are eligible for free meals). This policy, systems, and environmental approach to mitigating food insecurity provides potential for equitable obesity prevention but is one of many understudied policies from an implementation science and health equity lens.

Adoption of USM has demonstrated efficacy in reducing food insecurity, increasing student enrollment, positive health outcomes, and academic performance (9). Although this evidence-informed policy has the potential to significantly reduce risk of overweight and obesity, participation among eligible schools remains low. Currently, only 57% of eligible districts participate in the United States (10). In participating schools, organizational leaders (e.g., teachers, food service) still report challenges to promoting student participation in breakfast and lunch programming (6, 10). Further, although the Healthy, Hunger Free Kids Act stipulates nutrition requirements for all school meals nationwide, researchers have highlighted the disparities in the school food environment, such as lower quality of foods in low-income schools, predominantly serving Black and Latino students (11). The COVID-19 pandemic highlighted racial disparities in food insecurity and opportunities to address nutritional needs of underserved populations. While some districts successfully supported food access in Black and low-income communities during the COVID-19 pandemic (12), other studies found emergency school meal sites were somewhat incongruent with areas of high poverty and racial/ethnic minority populations, demonstrating a key gap in equitable implementation (13).

Successfully passing a policy does not guarantee it will be carried out as intended; without careful implementation, policies cannot yield their desired impacts. There is a lack of information regarding how school and district policies, such as USM, are implemented, warranting greater attention to the implementation determinants (i.e., barriers and facilitators), processes, and outcomes at multiple societal levels (i.e., students/families, school, local community, regional). The field of implementation science, which seeks to study and promote the systematic uptake of research evidence into policy and practice, provides pragmatic yet rigorous solutions to understanding if, how, and why policies are implemented, as well as potential strategies to improve implementation (14–16). The emergent focus on policy implementation science is emphasized as a means to address health disparities, since efforts to improve the adoption, implementation, and sustainment of public policies targeting underserved populations can increase their impact on mitigating health disparities (17, 18). By focusing on school and district policy implementation, researchers and practitioners can better understand how and why certain policies are implemented better than others, and the contextual factors that need to be addressed. Ultimately, this will build capacity in school systems to better serve the needs of students and families with a focus on health equity.

Numerous measurement tools have been developed to assess the quality of school health and wellness policies in the United States and internationally, but few have been developed for evaluating implementation determinants, processes, or implementation outcomes (often referred to as policy outputs) (19). For example, the US Centers for Disease Control and Prevention (CDC) School Health Index is a comprehensive assessment tool which can be used to evaluate the current school/district wellness policy comprehensiveness as it is written (20). A similar tool, the Wellness School Assessment Tool (WellSAT) developed by Schwartz and colleagues, was designed and validated to assess the quality and strength of policies (21, 22). Other tools similar to the School Health Index and WellSAT have been designed in Canada (23, 24) and countries across Europe (25) to help schools/district evaluate their own policies and plan for improvement aligning with federal mandates (8).

To ascertain the status of school health policy implementation measures, an international systematic review was conducted to locate and evaluate extant policy implementation measurement tools in literature published between 1995 and 2020 (19). Findings indicate that most tools were developed to assess one health policy topic (e.g., nutrition, physical activity, mental health), were developed by researchers without school/district partner or other practitioner input, did not report validity and reliability testing, and assessed only one or a small number of implementation determinants, processes, or outcomes. The systematic review also highlighted a lack of focus on health equity in existing measures that assess key determinants to, and outcomes of, successful implementation of policy which could potentially worsen health disparities due to inequitable adoption (19). As such, there is an opportunity to develop these measures with input from community partners and key implementation leaders so that their needs are placed at the forefront. To accomplish these goals, appropriate measures must be grounded in health equity.

### Project description

The overall objective of this project is to collaboratively develop a set of measures and metrics that can be used to study the implementation of school health policy through a health equity lens. School nutrition policy, specifically USM, will be the focal policy subject for these measures, but the products of this study will be designed in a way that they can be widely adapted to other health policies (e.g., physical activity, mental health, tobacco control, etc.). These tools will be adaptable to multiple health topics and interventions within the realm of school policy, with instructions for adaptation made available, thus providing a bank of shared measures for school-based researchers and practitioners to tailor to their work, enhancing the rigor of implementation science in schools.

This study has two primary aims:

- Identify key constructs related to equitable implementation of school health policies through a collaborative approach.
- Create measurement tools for key implementation determinants, processes, and outcomes and establish face and content validity through review of the health equity literature and rigorous community engagement techniques.

## Study design

**Figure 1:**
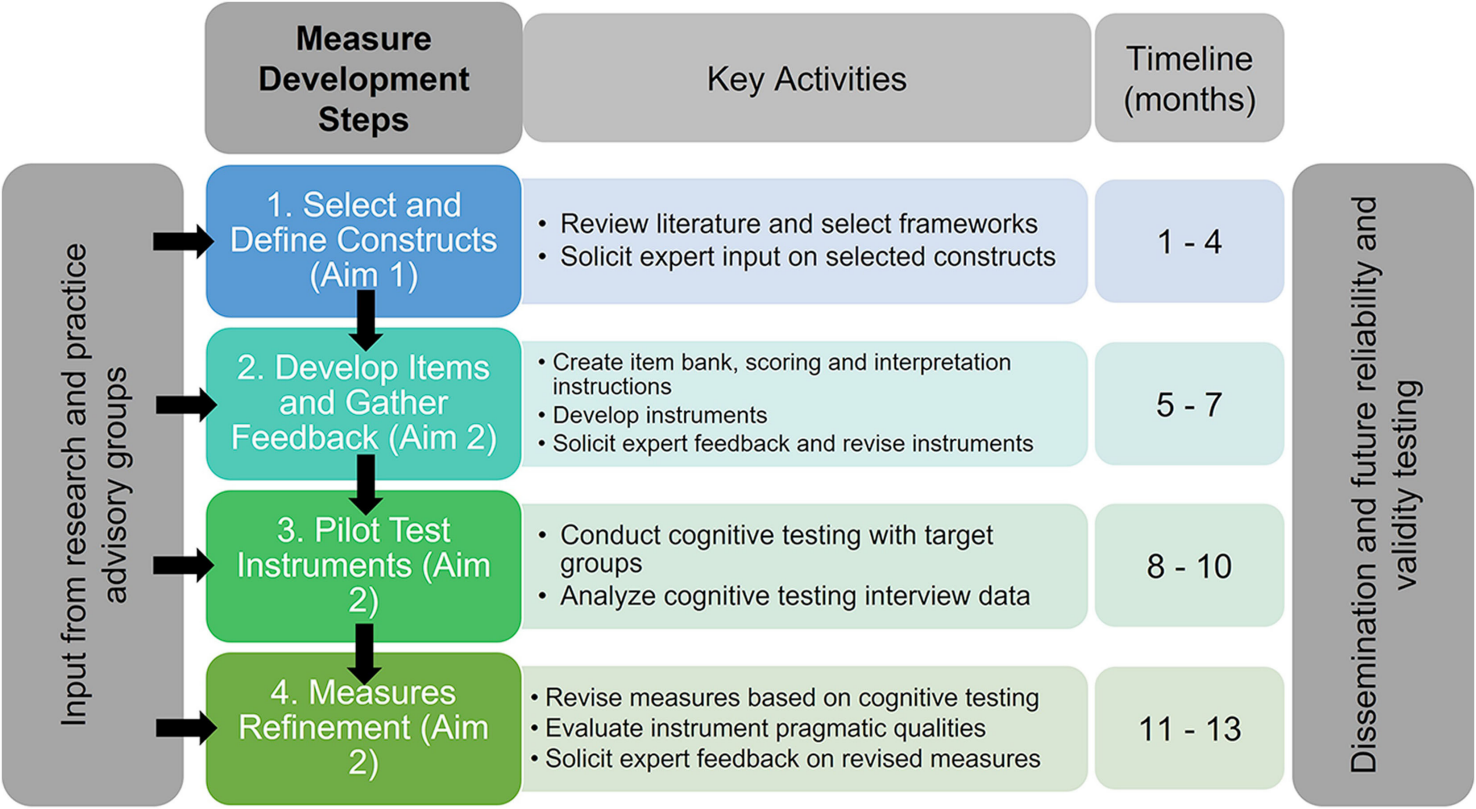
Overview of measure development study phases.

This measures development protocol will be driven by subject matter experts and community partner input and guided by established recommendations for development (26–32). This study follows steps similar to those taken in other measure development studies in the implementation science field to create items that can be easily modified to apply to a broad array of topics (33, 34). The current study will complete the following measure development steps: (1) identify and define constructs; (2) generate initial items; (3) pilot cognitive testing initial items; and (4) refine items based on cognitive testing. This pilot study is focused on the initial development stages and will inform future field testing and evaluation of psychometric properties, but these steps are beyond the current study scope. A visual overview of the study is provided in Figure 1. This protocol is registered with the Open Science Framework (osf.io/p2d3t) and approved as exempt by the Temple University Institutional Review Board (IRB number 29657); changes will be documented if deviations occur from the registration. This pilot study will take place on both a national and local scale; we will work with national organizations (described below) and the School District of Philadelphia (SDP). The SDP is the largest school district in Pennsylvania, serving over 200,000 students, 52% of whom are Black, 21% Latinx, 14% White, 7% Asian, and 5% Multiracial/other. All SDP schools provide breakfast and lunch at no cost to students because >40% are from low-income households. The SDP is a member of the Urban School Food Alliance (USFA), a US-based national organization that provides technical support to 17 of the nation's largest urban districts (>5,000 schools). Our team has worked to develop a strong partnership with the SDP and USFA through numerous meetings and projects; this relationship is critical to ensuring the success of the project.

### Identify project goals

We assembled an interdisciplinary team of scientists with expertise in health equity, school-based policy, and implementation science to conceptualize the study and parameters for the protocol. The team will meet regularly (i.e., every 2 weeks) to discuss the goals of the study and resulting products. In addition, given that the target audience includes researchers, practitioners, and community-based partners, designing for dissemination (35) to multiple audiences is a key priority from project onset through completion. Discussions will focus on how these perspectives can be solicited throughout the research process and drive much of the content and procedures.

This study seeks to develop qualitative and quantitative instruments to be deployed with several participant groups. Specifically, we will develop qualitative, semi-structured interview guides and quantitative surveys for use with students, parents/guardians, school principals, teachers, and food service staff and directors.

### Review of health equity frameworks for grounding

Upon confirmation of study goals and outputs, our team will review health equity literature compiled from ongoing review studies (36) and recommendations crowdsourced from experts in health equity research. From these, the research team will select health equity and implementation frameworks deemed applicable to the current project and its goals. These frameworks may include determinants (i.e., barriers and facilitators) influencing policy implementation, policy implementation processes, and/or outcomes, with particular emphasis on health equity (37). For the purpose of this study, we adopted the following health equity definition: “Everyone has a fair and just opportunity to be as healthy as possible. This requires removing obstacles to health such as poverty, discrimination, and their consequences, including [disenfranchisement] and lack of access to good jobs with fair pay, quality education and housing, safe environments, and health care. For the purposes of measurement, health equity means reducing and ultimately eliminating disparities in health and its determinants that adversely affect excluded or marginalized groups” (38). Given the novel and necessary integration of health equity frameworks in this work, we also realize that these frameworks will also bring constructs that reflect determinants of health equity and actions required to mitigate health disparities. As such, we refrain from confining frameworks to the three aforementioned groupings as the implementation and equity frameworks will converge over the course of this project. Specifically, the team will apply three key criteria (1) importance to health equity, (2) relevance to school policy, and (3) potential of constructs to be feasibly measured with practitioners, to decide whether frameworks should be included in the study. The goal of these criteria is to enhance discussion and deliberation, and to avoid saturation and/or duplication among the frameworks.

Upon selection of these frameworks, we will review each article within a worksheet which will document (a) the setting/context(s) to which the framework is applied; (b) key framework constructs and relevance to school-based policy; (c) levels of conceptualization (e.g., individual, organization, community, policy, system); (d) most salient framework type (i.e., determinant, process, outcome, or a combination thereof); and (e) any associated measures or resources within the article text. After this process, we will arrive at a completed “matrix” of characteristics to inform key decisions about framework suitability. The goal of this review will be to determine a concrete set of frameworks which will guide the conceptualization of health equity and related concepts and inform measures development, with particular attention to how health equity frameworks can inform selected implementation science frameworks. The investigative team will review the matrix of framework characteristics and generate consensus on which to include to inform the measure development. The following sections describe each of the four measure development steps of this project.

### Select and define constructs

Based on the results of our screening and review, pertinent frameworks will be selected to ground the development of policy implementation measures. The framework matrix will provide a list of key constructs and definitions from health equity and implementation frameworks which we will map on to one or more determinants, processes, and outcomes measures to be developed (see Supplementary Table 1). We will refer to supplemental literature in the event key constructs are not adequately defined in the frameworks. The research team will meet to discuss these constructs and how they align with school policy, and the potential of these constructs to be applied to other policy topics.

#### Subject matter expert and community leader feedback on selected constructs

Once a comprehensive set of constructs has been established and defined, we will solicit input from key informant groups to ensure the constructs align with their priorities and that relevant and important constructs are not missed. We will develop a brief survey, which will provide the goals of the project and include an initial list of constructs, their definitions, and examples items that could be included in a survey or interview guide. The survey will ask respondents to rate the importance of each construct to equitable policy implementation in schools and will include space for respondents to provide other factors or resources they deem important to consider.

We will disseminate the survey to several subject matter expert groups and organizations including researchers and practitioners from the Nutrition and Obesity Policy Research and Evaluation Network (NOPREN) school wellness working group funded by the CDC and members of the USFA (this group is funding this study). The USFA comprises 17-member districts from across the nation and its members work predominantly in school food service. Their perspectives of these constructs will provide pragmatic feedback on how they relate to their implementation experiences and insights on what should be assessed in measures. We will also invite the SDP equity audit working group, comprised of multiple roles (e.g., administration, teachers, food service, counselors, etc.) working toward integration of health equity into the districts' metrics and processes. Feedback from SDP as a key partner in this work will provide integral support and knowledge to help refine these constructs and definitions which reflects their needs and interests. We also will disseminate the survey to members of the Society for Implementation Research Collaborative, School Nutrition Association, and the Temple Office of Community Engaged Research and Practice, among other local/regional organizations which specialize in community engagement. All respondents will have the option to enter a random drawing to win one of twenty $25 gift cards in appreciation for their contribution.

After each round of feedback, the research team will meet to refine this list of constructs and their definitions. Upon selecting the final set of constructs, the team will generate items to measure each of these constructs.

### Item development and feedback solicitation

We will develop initial drafts of interview guides and surveys to assess key policy implementation determinants, processes, and outcomes through an equity lens. Our goal is to build from existing measures in the implementation science, public health, and health equity literature, creating new items when existing sources do not evaluate constructs of interest. We anticipate our study will result in one survey and one interview guide for each participant type with supplemental item banks, but will remain flexible to feedback we will receive in Aim 2. Similar to the steps taken by Lewis et al. (34), the research team will review example measurement tools, published measures reviews, and online measures repositories to determine whether existing instruments contain relevant items or scales that align with the conceptual content of the selected constructs and can be used verbatim or adapted for use in the new measures under development (19, 39–46). For each chosen construct, we will draw upon relevant literature and examples from existing instruments to draft items. The research team will review items for consistency with the construct definition, relevance to the project topics and goals, clarity, and conciseness.

To ensure the measures are practically applicable, we will apply the pragmatic rating criteria from the Psychometric and Pragmatic Evidence Rating Scale (PAPERS) (30), which rates measure properties on a scale of−1 (poor) to 4 (excellent) on five criteria. In the item development phase, we will use the Flesch-Kincaid readability test in Microsoft Word to ensure items are written at a reading level appropriate for the target respondent population (47). For the student-facing measures, we will aim for measures written at a 6–7th grade reading level; for adult-facing measures, we will aim for a 9–10th grade reading level, achieving a good (3) to excellent (4) rating on the PAPERS. All measures will have fewer than 50 items, rated as good (3) on the PAPERS length criterion. The other PAPERS criteria – cost, ease of training, and ease of interpretation will be used to guide the dissemination of final measures such that they are freely available and have adequate information for administration and scoring.

The research team will maintain an item bank that documents the construct assessed, theoretical foundations, whether the item was adapted from an existing instrument or team-developed, the item wording, item response options and anchors, instructions for coding items and subscales, and instructions for interpretation. For items adapted from existing instruments, we will record the original source citation, how the item was adapted, justification for the adaptation, and available psychometric evidence from previous uses.

Before conducting cognitive testing, we will solicit feedback from the same sample of NOPREN, HER, and USFA network members who provided construct feedback to ensure familiarity with the study and its overarching constructs. This will be via a follow-up survey which provides items linked to constructs and a rating scale of clarity and fit within the current study. Respondents will also have the option to provide feedback through open response questions, and to provide suggestions on additional items/questions. Data will be analyzed on a per-question basis and will facilitate preparation for pilot cognitive testing.

### Pilot cognitive testing

The research team will pilot the instruments with representatives from each of the participant groups and refine the instruments prior to collecting pilot quantitative data to evaluate preliminary psychometric properties (i.e., validity and reliability). The goal of cognitive testing interviews is to determine if questionnaire items are readily understood by target respondents and work to assess intended constructs. Cognitive testing is a best practice in measure development, and is typically conducted prior to field testing instruments (48, 49).

#### Recruitment and sampling

We will conduct non-random, purposive sampling to achieve representation of diverse demographic and school setting characteristics. We will recruit participants through NOPREN, USFA, SDP, and other community-academic partnership networks. We will work with a university partner through a Patient-Centered Outcomes Research Institute-funded engagement grant to conduct cognitive testing with high school students from across the state of Pennsylvania. Participants will include students, parents/guardians, teachers, principals, and school food service staff. We will recruit approximately 10 participants from each group; small sample sizes are appropriate for cognitive interviewing (31). Participants will receive a $20 gift card incentive for participation.

#### Cognitive interviewing procedures

The research team will develop cognitive testing procedures and will be trained in cognitive interviewing techniques by a research team member experienced in cognitive interviewing, qualitative methods, and measure development. The research team members conducting cognitive testing interviews will be experienced in qualitative interviewing. The interviewer instructions will include distress procedures in the event of an adverse event. Participants will receive brief information about the interview goals and procedures during recruitment and the interviewer will explain the purpose of the cognitive interview to the participants at the start of their interview session.

Following procedures used by Bobrovitz et al. (50), half of participants testing the survey instruments will receive this prior to the interview to complete and the other half will complete the survey at the start of the interview while the interviewer is present. Participants will receive a copy of the instrument(s) prior to the testing session and will be instructed to review the instrument and note any questions or comments they have on the questionnaire instructions, items, and response options. For respondents completing the survey during the interview, the interviewer will track how long the participant spends reviewing the instrument and responding to the items. All participants will be asked to return their completed instrument to the research team.

Once participants review the instrument, the interviewer will follow a semi-structured interview guide that combines think aloud procedures and verbal probing (31, 32, 51). The interviewer will instruct the participant to read through one item at a time, summarize in their own words how they interpret the question, and verbalize their reactions to the item (e.g., approval, confusion, discomfort). Interviewers will probe participants to provide further detail and invite participants to provide recommendations on specific item wording or overall questionnaire structure. Example interview questions include:

- What reactions did you have when you read this question?
- In your own words, please describe what you think this question is asking.
- What does the term ___ mean to you?
- How confident are you that you would be able to answer this question on your own?

Interviews will be audio recorded with participant permission. Interviewers will be trained to look for non-verbal cues from participants and will keep field notes of non-verbal reactions and observations not captured in the audio recording.

Interviewers will debrief regularly with the research team. As cognitive testing is an iterative process, the research team will decide whether clear needs for revisions to the interviewing procedures or the instruments undergoing testing emerge after the first several interviews and will revise these materials for subsequent interviews as necessary. The stopping criteria for cognitive interviews will be when our analyses reveal saturation in themes (e.g., no new feedback on items, feedback converges). As others have previously noted, cognitive interviewing sample sizes may not be sufficient to achieve complete saturation and stopping criteria may operate under a principle of diminishing returns (52–54). In cases where feedback conflicts or new feedback is of minimal value, we will discuss items as a team and consult additional experts as needed to make a decision on continuing additional interviews.

#### Data management

All study materials and interview data will be stored in a secure location. Interview audio recordings will be transcribed using a professional transcription service. Transcripts and coding documents will be anonymized (i.e., participant names and identifying information such as place of work will be replaced with participant ID numbers and generic descriptions). Interviewers will create interview notes using a standardized template, which will include participant information (e.g., participant ID, gender, age, education level, respondent group), summary of the original instrument (including item responses and participant notes), and interviewer observations (32).

The research team will develop a coding matrix to organize data for each instrument (32). Each matrix row will represent a participant, and each column will represent an instrument item. Additional columns will include participant background information, data for instrument instructions, and general reactions and findings. Coders will review each interview transcript and the interview note templates and enter data for each item into the coding matrix.

#### Data analysis

Once the interview data are entered into the coding matrix, two coders will conduct descriptive and explanatory analysis (32). The descriptive analysis allows for understanding how measure items were interpreted and how answers were formulated. The explanatory analysis serves to identify how instrument instructions, items and response options should be revised (e.g., reword, condense, or expand response options), if and how the instrument should be restructured (e.g., reorder items or subscales), or whether items should be eliminated.

The research team will develop an a priori code list, and coders will add inductive codes that emerge from their review of the data. Coding will be conducted using a dual non-independent approach in which a primary coder reviews the matrix and applies codes, and a second coder checks the coding for accuracy and completeness. The secondary coder will note any disagreements, which will be discussed by the research team to generate consensus.

In the descriptive analysis phase, coders will apply codes to classify how items were interpreted by participants, strategies used to answer each item, acceptability of the question, and to identify problems with an item. In the explanatory analysis phase, coders will seek to determine reasons for identified problems and to identify patterns across respondents. In the final step of the explanatory analysis, coders will seek to identify implications of the problems identified. The coders will create coding summaries for each instrument. The research team will discuss the summaries and will make decisions to revise the instrument based on the findings.

The team will also generate summary statistics of the completed survey instruments and will review response distributions for patterns of concern (e.g., neutral response bias, extreme response bias). The sample size for cognitive interviews for each instrument are insufficient to conduct formal quantitative tests at this phase, but may yield trends to evaluate in subsequent field tests.

### Measures refinement

Analysis of feedback from the cognitive interview stage will guide refinement of measurement tools by the research team. This is a critical step in establishing face validity among researchers and practitioners. Initial refinements will prioritize the feedback from practitioners given the pragmatic nature of this research and the tool usage. The research team will also begin to develop user guides and instructions for adaptation to other policies (e.g., physical activity, mental health) to facilitate their usage.

Our team will send a survey to the groups involved in providing initial input on construct prioritization to solicit their feedback on pilot quantitative measures. Representatives from each target respondent group (e.g., school staff) will be invited to complete the quantitative measure via an online survey and to provide brief feedback on individual items and the instrument as a whole. We anticipate receiving 20–30 responses per target group in this feedback round. The team will generate descriptive statistics to examine the response characteristics (e.g., response distribution). We will calculate initial internal consistency on multi-item subscales using the reliability command in SPSS software. We will review feedback on the items to determine if additional refinements are warranted before larger scale pilots to evaluate psychometric properties.

### Pragmatic measures properties

A key barrier to using validated tools is often the length (i.e., number of items), lack of information on training and interpretation, and their cost to use. To expand use of validated measures within implementation science and school policy, it is therefore imperative to report the pragmatic properties of tools as a part of dissemination. The PAPERS (30) provides a much-needed scoring procedure for improving transparency in measurement tools in implementation science. The PAPERS scale offers objective, standardized rating criteria on five pragmatic qualities: number of items, readability, cost to use the instrument, assessor burden (ease of training), and ease of scoring and interpretation. The PAPERS scoring criteria have been applied in previous work by the study team (19) and will be applied in the same way such that higher numerical scores for each criterion indicate more pragmatic and usable tools. Given the scope of this project, we will examine and report the pragmatic properties of all measurement tools, with the view toward examining psychometric properties in subsequent research.

### Research team positionality

Given the nature of this work, we believe it important to reflect on and pose our positionality and how it shapes the perspectives we bring to this project. Reflexivity informs positionality, thus we take a reflexive approach to self-assess our views and positions and how these may direct the design, execution, and interpretation of this study and its results (55). As researchers, we must recognize the positions of power and privilege we hold and their impact on each aspect of this study. Such reflexive process takes time and patience, and the understanding that a positionality statement is fluid and may change over time as researchers become more embedded in their work (55). Below we provide such statements for each author and how their positionality shapes their role in this project.

Dr. Gabriella McLoughlin (she/her/hers) is a licensed K-12 teacher and a first-generation college graduate. She has lived experience of food insecurity, overweight/obesity, and fluctuating household income; these experiences fueled passion and motivation toward addressing issues of hunger and food insecurity in youth. She is also passionate about supporting school-level initiatives to build and sustain health promoting programs, and constantly approaches issues from a practitioner standpoint. She identifies as white and cis gender with no physical or intellectual disabilities, which also represent positions of power within society. These positions provide a privileged viewpoint and may influence the design, execution, and interpretation of this study. Accordingly, it is imperative to constantly reflect on each decision regarding study design and development of partnerships, ensuring that a true collaborative approach is adopted with local school districts and organizations, and that their voices are equitably reflected in each part of the research process.

Dr. Chelsea Singleton (she/her/hers) is a nutritional epidemiologist who has studied social, political, and environmental determinants of poor diet and obesity for the past 10 years. She identifies as an African American cis-gendered woman. She was raised by a single parent in an economically disadvantaged community with limited availability of healthy food. That experience inspired her to research structural barriers to healthy eating in low-income communities of color. Nevertheless, Dr. Singleton has received several opportunities to advance her education. She currently works as faculty at the most expensive private research institution in Louisiana. Her identity (gender and sexual orientation), educational background, and place of employment have afforded her privilege and positions of power, which may influence how she designs studies, navigates community-academic partnerships, and understands the meaning of “health equity.”

Dr. Lindsey Turner has studied and applied the principles of community-based participatory research over the past 25 years in her professional work as an academy-based researcher. As contingent non-tenure track faculty she works from an unprotected position, within an institutional system that has historically held power and privilege. She holds identities that confer privilege including being a white, cis-gender female without intellectual or physical disabilities. Her primary identities include her status as a mother, an immigrant, and a prevention scientist. She has lived experience with chronic physical and mental health issues that fuel her passion to promote school environments that are nurturing for all children and adolescents.

Ms. Callie Walsh-Bailey is an MPH-trained research assistant and public health PhD student. She is a first-generation college graduate from a socioeconomically disadvantaged household. Her background living in underserved rural communities, experiencing housing and financial insecurity, un- and under-insurance, and providing care for family members with complex health needs motivated her pursuit of health equity-focused research. She identifies as a queer, cisgender woman who experiences a chronic health condition. As a white, US citizen graduate student at a private research university, she holds a privileged social position. As a student, she holds a relatively vulnerable professional position. Her intersecting identities afford varying power or vulnerability and influence her conceptualization of health equity, recognition of research priorities, and how she interfaces with and is perceived by groups and communities involved in and potentially affected by her research.

Collectively, this interdisciplinary research team identifies their motivations and passions toward equitable implementation of policies designed to reach those most at risk for food and nutrition insecurity, obesity, and other chronic diseases. It is important to acknowledge this viewpoint and to make the distinction between policy and political views. We must also recognize our role as curators of information and knowledge, not creators. We seek to honor those who have worked for years in health equity research and to lift their work up by applying it to the current implementation context.

## Discussion

This protocol describes key first steps in developing pragmatic, rigorous, equity-focused measures of school policy implementation. Following pilot cognitive testing, we will analyze the data and refine measures to ensure reflection of participant voices. Further, we will develop a measures repository in collaboration with NOPREN, HER, and USFA with the goal of expanding reliability testing with practitioners, community members, and students. Through this, we aim to pilot surveys in future studies to establish reliability and validity (e.g., convergent validity, discriminant validity, structural validity, responsiveness, and norms) (30). Given this is an initial measure development, it will not be possible to assess known groups, predictive or concurrent validity at this stage.

This study's strengths include the use of established measure development best practices (27, 28, 39), engagement of key partners, including target end users, in each development stage, and transparent reporting of early findings so that they may be used by other researchers and practitioners (56). This study contributes to filling a gap in the measure development methods literature in that we provide detail on processes for identifying relevant constructs and engaging target audiences in developing and refining measures often not detailed in the literature. This provides steps that other researchers and measure developers can emulate. Although the ultimate goal of this work is to develop quality measurement tools that can be adapted to an array of measurement topics, this preliminary work will focus mainly on USM as the policy topic of interest. As this is an initial development pilot, we use non-random sampling methods to acquire collaborator input. This study may have limited generalizability beyond the involved participant groups, though establishing generalizability is not a central goal at this current phase.

We anticipate that this project will have a positive impact on how we conceptualize and investigate health equity in the context of policy implementation. One of the many challenges in this work is the ambiguity regarding how we assess health equity and its inclusion as part of the research process (57). By developing measures (both qualitative and quantitative) for assessing issues related to equity in our implementation determinants, processes, and outcomes evaluations, we will facilitate detection of factors that may disproportionately benefit access to interventions in some populations while systematically excluding others. This will provide researchers with a means to identify these issues, whether structural, interpersonal, or cultural, and adapt/modify their intervention delivery to better meet the needs of their target population. This project marks an initial step in advancing use of health equity in implementation science for advancing overall population health.

## Ethics statement

The studies involving human participants were reviewed and approved by the Temple University Institutional Review Board. Written informed consent from the participants' legal guardian/next of kin was not required to participate in this study in accordance with the national legislation and the institutional requirements.

## Author contributions

GM conceptualized the study. GM and CW-B designed the study procedures. CS and LT provided input on the study conceptualization and design. GM, CW-B, CS, and LT contributed to the writing and editing of this manuscript. All authors have read and approved the final manuscript.

## Funding

This study is funded by the Urban School Food Alliance (USFA). USFA affiliates are invited to provide input on the measures developed through this pilot.

## Conflict of interest

The authors declare that the research was conducted in the absence of any commercial or financial relationships that could be construed as a potential conflict of interest.

## Publisher's note

All claims expressed in this article are solely those of the authors and do not necessarily represent those of their affiliated organizations, or those of the publisher, the editors and the reviewers. Any product that may be evaluated in this article, or claim that may be made by its manufacturer, is not guaranteed or endorsed by the publisher.

## Author disclaimer

The views expressed in this manuscript represent solely those of the authors and do not necessarily represent the USFA.
