# Supplementary material for: Investigating implementation of school health policies through a health equity lens: A measures development study protocol
Source: Front Public Health. 2022 Nov 30;10:984130. doi: 10.3389/fpubh.2022.984130 (PMC9747935; doi:10.3389/fpubh.2022.984130)
Supplement: Supplementary file 1 [file Table_1.pdf]

Factor	Adaptation to School Policy/Equity Context	Example item(s)	Not at all important	Somewhat important	Neutral	Rather important	Very important	Not sure/NA
			1	2	3	4	5	0
Socioeconomic, Cultural and Political Context	The socioeconomic, political, cultural, and functional mechanisms through which a society operates. Includes government apparatus (e.g., federal dept of education, state legislatures), political traditions, financial institutions, corporations, labor markets, citizens' legal rights and obligations, and sociocultural values and norms, etc. The socioeconomic and political contexts determines the availability, distribution, and quality of public infrastructure and resources (e.g., public schools, financial, non-monetary, physical infrastucture and staffing resources within schools). The sociocultural context shapes the nature of social relationships and values attributed to health (e.g., policy actor networks responsible for operationalizing universal school meal policies at the local level and their value or prioritization of equitable access to school meals)	Items for socioeconomic, cultural, political as separate factors. Political: To what degree do political officials (i.e., local politicians, mayors, city officials) support the policy and its implementation? Socioeconomic: To what extent do local funding policies (e.g., tax laws, school funding mechanisms) impact policy implementation at your school? Are these policies affecting communities of color, low-income communities, or other marginalized communities? Can you provide an example?						
Health Policy Context	School meal policies, programs, and resource allocation affect the presence, location, and organization of education and food service physical infrastructure, the provision of supplies and services, staffing, and human resource management. Protocols and guidelines for carrying out school meal policies designed with equity at the forefront may ensure: 1) services are respectful and responsive to students' and families' needs and preferences (Acceptability); 2) there is a balance of evidence-informed practices with student and family needs and preferences for appropriate services; 3) service delivery prevents and minimize food insecurity risks; 4) use of best and updated scientific knowledge and practices for optimal outcomes for students and families	To what extent does the policy more driven by evidence, family needs and preferences, or neither? To what extent do you think this policy aligns with family needs?						
Social Stratification Process	The ways a society, community, or organization is hierarchically stratified, based on systematically unequal distribution of power, prestige, and resources, as well as discrimination. Social stratification indirectly determines differential health-related exposure, vulnerability, and consequences. The reduction of health inequities requires micro- and macro-level changes to a more equal distribution of power (e.g., more agency given to students and families in decision making)	How is decision-making power distributed across your school system? for parents: how much of a say do you get in your child's meal program at school?						

Social Location	Rank or position an individual is attributed to hold in a sociocultural and economic hierarchy within a society, community, or organization at a given time. This relational position is shaped by the interacting, intertwined influences of power relationships, access to resources, prestige, and discrimination. Social Location can be measured through power-related measures (e.g., who within schools holds what level of decision-making control, household authority, gender roles), resources-based measures (e.g., income level and social class of students and families), prestige-based measures (e.g., educational achievement and occupational status), and discrimination (e.g., immigration status and religion)	What kind of decision making power do you hold within your family/school/community? Do you hold any priviledged positions that may impact your status and ability to make executive decisions?						
Material Circumstances	The financial means (income and material or intangible assets) allowing purchase and consumption for ensuring healthy, dignifying living conditions. Material Circumstances include resource-based measures related to (a) satisfaction of basic needs (e.g., food security); (b) possession of household or organizational amenities (e.g., refrigeration to store perishable foods); (c) potential consumption of social goods and services, including education. Material Circumstances can directly affect health-related behaviors (e.g., the high cost of vegetables and fruits may lead low-income familes to consume low-cost processed foods)	What would you say are the resources you have at home to purchase and prepare food? What resources are you lacking that may enable you to prepare high quality meals?						
Social Circumstances	Social circumstances includes several concepts of social cohesion and social capital. Structural social capital involves the extent and intensity of social connections or engagement in network activities (e.g., parent involvement in PTA, school board meetings to share in decision making around how school meal policies are carried out). cognitive social capital refers to perceptions of trust, solidarity, and reciprocity, and social support (e.g., food service staff, administrators have trusting working relationships and support eachother in meeting student nutrition needs). Bonding capital refers to tangible or intangible resources that people with the similar social background can access through their participation in social networks (e.g., students with shared ethnic backgrounds share foods, shape social norms and attitudes towards school meals).	What would you say is the degree of trust between the school nutrition staff and the rest of the teaching staff? What about between the students and staff? How do you think these dynamics influence how (policy) is implemented?						

Environment	Area-based measures and physical and social features of a space (e.g., a school, a neighborhood). Also encompasses the concept of place: a combination of the physical characteristics, context, and meanings attributed to the space where people live, work, or play. Context includes the absence or presence of environmental tangible and intangible features, including natural (e.g., climate, geographic landmarks), built (e.g., neighborhood grocery stores, school cafeteria rooms), and sociocultural features of the environment (e.g., community's reputation, graffiti)	Cross-list with CFIR structural characteristics? Example item for school staff: What would you say are some of the school's physical characteristics that may influence implementation of the (policy)? Size of building, structure, layout, condition? What about the surrounding area, such as crime, safety, proximity to public transport?						
Health Beliefs	Individual or collective perceptions of what influences health in a positive or negative way. Health Beliefs are a key component in the way people rationalize the benefits or harms of adopting and maintaining certain health behaviors (e.g., avoiding highly processed, energy dense foods to lower risk of cardiovascular disease).	item for all: What are your beliefs toward (policy)? How do you think it might promote health in students/children?						
Psychosocial Stressors	Any social, environmental or external challenge that requires an individual to adapt to it. These stressors can be acute (e.g., a recent life event such as job loss) or chronic (e.g., continuous daily discrimination based on race or sexual identity). A policy-related psyhcosocial stressor may include social exclusion due to a policy that discriminates against or does not accommodate certain groups or attributes (e.g., provision of meals does not meet dietary needs of certain religions)	What are some issues that cause stress related to (policy)/accessing high quality food and nurition? To what extent do you feel discriminated against based on your race/ethnicity, income status, sex/gender, or other identity when it comes to accessing (policy)/healthy food?						
Need	self-perceived or professionally evaluated Need to utilize health-promoting resources, such as nutritious foods	To what extent to you feel you need access to nutrition assistance? (family) What is the extent of the need for school meal programming within your school? how much of a difference does this policy make? (school staff)						
Utilization of health-promoting resources	Use (or lack thereof) of health-promoting resources among students and families, provided by the public, private, and non-for-profit sectors.	(schools) Do you know if students/families at your school are participating in other assistance programs such as SNAP/WIC, TANF, or other programs? (families) what other programs are you participating in now? Are you finding them helpful in supporting your ability to afford healthy foods?						
Innovation source	trust in gov't (or other entity mandating policy) & power differential b/wn policy source, implementers, and recipients/end users	What had you read or heard (social media, other sources) that made you think (policy) could work for your school?<br>How did this knowledge affect your perception of the policy? Other staff?						

Innovation Evidence-Base	perception of evidence quality/credibility among implementers, end users; with whom, by whom is evidence created	What do you know about the evidence used to support this policy? To what extent do you think the researchers are trustworthy?						
Innovation Relative Advantage	is policy better at achieving nutrition equity than previous approaches	Do you think this policy will help you better achieve gains in student health and equity than what already exists?						
Adaptability	can/does policy align with local schools, recipients	How might you need to adapt the policy to fit the needs of your students, parents, and staff?						
Innovation Complexity(CMPX)	how many stakeholders are needed, is it comprehensive (how many areas of the school)	How complex is the policy to implement?						
Unanticipated Events	Large-scale unanticipated events(e.g., pandemic, flood, largess in funding)	How might (COVID/other natural distaster) shape how you implement the policy? Have you had to make changes to other policies due to the ongoing pandemic?						
Partnerships & Connections	Networks and relationships between the school and external entities (e.g., school boards, neighborhoods, community non-profits, etc.)	How might you partner with the district/other organizations already working with your school to implement this policy to meet the needs of students/families?						
Structural Characteristics	Physical and social architecture, age, maturity, and size of a school	Does the school have a longstanding relationship with the surrounding community? How big is the school and how does its size impact policy implementation?						
Relational Connections (networks & communications)	Nature and quality of formal and informal relationships within and across structural, professional ,or other boundaries within the school; nature and quality of formal and informal information sharing within a school	Can you describe your working relationship with administration? To what degree do they prioritize policy implementation and health equity? Your supervisor? Supervisors of other colleagues?						
Culture	Collective beliefs and philosophies of those within the school setting related to school policy implementation	How do you think your school culture (general beliefs, values, assumptions that people embrace) affected the policy implementation? Can you describe an example that highlights this?						
A. Person-Centeredness	values, beliefs,and norms around caring, supporting, and addressing the needs and welfare of the people delivering the policy (e.g., teachers, food service staff) and/or recipient (e.g., students)	To what extent do you feel that those in your school care about each other and their wellbeing? What about that of its students?						
B. Leadership Commitment	leaders, managers, and/or administrators are involved and provide visible support for implementing the policy	How committed is your school administrator(ion) in implementing this policy? Can you give an example of this?						

C, Relative Priority(RP)	the policy is important to implement compared to other initiatives	Where does this policy fit in terms of priority? How does it compare with other initiatives that are ongoing/addressing similar areas of school health?						
Available Resources	Perceptions of the degree to which there are sufficient resources within the school (e.g., funding, staff, physical space, equipment, other materials, time) dedicated to implementing and delivering the policy and how this may influence implementation success or failure.	How much attention/time/training is being given to this policy? Is this siffucient? What are some resources that you need for better and more equitable implementation?						
Characteristics of individuals	Qualities and nature of individuals (e.g., food service staff, principals) involved in implementing or delivering the the policy (e.g., knowledge, skills, intentions, etc.)	Who were the key influential individuals to get on board with this policy?<br>What are these stakeholders saying about the policy? (this could have been other teachers, parents, students, and outside stakeholders who are influential in the school setting).<br>To what extent did they influence others' use of the policy? The success of the implementation?						
A. Implementation Leader(s)	the individual(s) who lead(s) or champion(s) efforts to implement the policy (e.g., leaders among food service staff, school administrators)	Who leads implementation of the policy? Is it mostly you or did you work as a team? How did your group come into this role? Appointed? Volunteered? Voluntold? What attributes or qualities do you feel made you the best leader(s) of this program? Does this person have sufficient authority to do what is necessary to implement the intervention?						
B. Implementation Team Members	the individuals (e.g., staff, administrators) who actively participate in or support theimplementation team, including people responsible for delivering the policy and recipients (parents, students) representing their broader peer group	How far reaching is the implementation team? do they represent diverse areas of the school system and backgrounds (i.e., racial/ethnic minority, other marginalized identities)?						
C. Opinion Leaders	the individuals who influence the attitudes and beliefs of their colleagues (e.g., well respected teachers, food service staff)	Who are the leaders within your school and how do they feel about the policy? Are there differences among teachers/staff according to race/ethnicity, "status" (i.e., hierarchy) or other factors?						

D. Advisors/External Change Agents	may include subject matter expert groups, student or parent advisory boards	How involved is your school's parent advisory panel? What is the dynamic between your school and this group? How might this impact implementation? are you collaborating with any activist groups who are passionate about health equity? if not, can you think of other outside groups that would be helpful? Talk about how this may influence implementation						
Increase Healthy Options	Policies/strategies that provide optimal access to engage in health behavior such as nutrition, physical activity, social and emotional wellbeing, etc. Presence of supports which encourage this within school policy and programming.	Policies/strategies that provide optimal access to engage in health behavior such as nutrition, physical activity, social and emotional wellbeing, etc. Presence of supports which encourage this within school policy and programming.						
Reduce Deterrents to Healthy Behaviors	Intentional efforts to reduce barriers to engaging in health behavior/nutrition assistance programming. Mitigating stigma, discrimination, issues of safety and feasibility of access	Intentional efforts to reduce barriers to engaging in health behavior/nutrition assistance programming. Mitigating stigma, discrimination, issues of safety and feasibility of access						
Improve Social and Economic Resources	Meaningful integration of nutrition assistance programming, partnering with local SNAP/WIC providers, food banks, and other poverty-alleviating systems	Meaningful integration of nutrition assistance programming, partnering with local SNAP/WIC providers, food banks, and other poverty-alleviating systems						
Build Community Capacity	Empowering students and families through meaningful engagement and involving them in decisions about school policy such as USM/CEP implementation. Developing partnerships with other community organizations that may help with logistical aspects of implementation.	Empowering students and families through meaningful engagement and involving them in decisions about school policy such as USM/CEP implementation. Developing partnerships with other community organizations that may help with logistical aspects of implementation.						
Meet basic food needs with dignity	Understanding that individuals have side hustles, and often need more money to meet basic food needs. Domain also includes emergency food assistance and the reinforcing loops stigma and stereotypes. Fundamental need for food to sustain life and highlights how stigma can delay access to food assistance. Values of freedom, agency, and dignity must be prioritized	How much agency do you have regarding access to government assistance programs such as SNAP/universal free meals? Do what degree do you feel empowered to participate in (specific policy activity)?						

Supply and Demand for Fresh and Healthy Foods	Includes feedback loops such as healthy food retail, job security, and food culture and norms. Store owners need to be motivated to sell fresh foods at market value. This is influenced by motivation of food store owners to supply fresh and healthy foods through market-based models, which is influenced by local food distribution infrastructure as well as consumer demand. This feedback mechanism is moderated by neighborhood investment for racial equity, such as lending strategies that offset operational costs for stores. Two reinforcing loops related to job security (R3) and food culture and norms (R4) reveal interdependencies between growth in supply of fresh and healthy foods with demand-side factors such as household financial capacity and food preferences.	cross-list with GTE Increase Healthy Options: To what degree do you feel that your local neighborhood is invested in health and racial equity? How is the demand for affordable healthy foods met with the supply? Is what's offered compatible with						
Repair	Assess experiences, attitudes, behaviors, and beliefs of disparity populations about the institution that have roots in the past, and may have bearing on willingness of or ability to engage with institution	How much trust do you have in the ability of (school) to meet you and your familie's needs regarding food insecurity(other policy issue)? Has anything happened to change this level of trust over time? For schools: how much trust do you have in (university collaboration) and their ability to help you with implementation of (policy)?						
Restructure	Assess structures in the organization that maintain systematic exclusion of disparity populations; or provide advantage/ privilege to others at the exclusion of disparity populations (Sources of "insults"; structures that continue to create risk for some populations)	What are some potential ways that systems or policies in your school may unfairly disadvantage those who are from diverse populations? Can you think of an example?						
Remediate	Assess needs for protection of individuals in disparity populations against existing insults, protections that need to be in place until the insult can be structurally removed	(if there are disparities impacting diverse/underserved populations) How can this be improved or mitigated through policy						
Remove	Identify Structures, attitudes, beliefs, practices or experiences specific to "Race/ethnicity", low SES or gender that confer disadvantage to these populations	(extension of previous item)						
Acceptability	perception among school staff, students, and families that a given school meal policy is agreeable, palatable, or satisfactory, particularly for students who experience food insecurity	Do you agree that (policy) is a good solution to addressing (hunger/other outcome)?						
Adoption	the intention, initial decision, or action to try or employ a school meal policy or practice associated with a school meal policy (e.g., ??)	Does your school have any written policies, procedures, or guidelines that address the following specific nutrition qualities of food and beverage items sold or served (or other health policy target)?						
Appropriateness	perceived fit, relevance, or compatibility of the school meal policy for a given school setting, school staff, or students & families; and/or perceived fit of the policy to address a particular issue or problem related to health equity	How appropriate do you think universal school meals (other policy target) is for your school in addressing nutrition insecurity (other policy outcome)?						

Feasibility	the extent to which a school meal policy can be successfully used or carried out within a school setting to advance health equity	How easily do you think schools can obtain culturally appropriate foods that meet the nutrition standards set forth by the policy? Or can successfully meet the nutrition (other policy outcome) needs of students? What complexities are involved with implementation that need to be overcome?						
Fidelity/Compliance	degree to which a school meal policy was implemented as mandated or as intended by policy makers and advocates for the policy	How well is (school meal program) being implemented and to what extent is this being implemented according to federal nutrition requirements?						
Reach/Penetration	integration of the school meal policy and related food service practices within a school	What is the proportion of students who participate in school meals relative to the school population?						
Sustainability	the extent to which a school meal policy is maintained or institutionalized within a school's ongoing, stable operations	How sustainable do you think (school meals) policy is withiny your setting?						
Cost	the financial cost of implementing a school meal policy, including costs of delivering meals as mandated, costs of resources and staff needed to implement the policy	Overall what are the costs associated with implementing (policy)? Is this policy financially feasible within the current budget structure?						
