# Supplementary material for: Investigating implementation of school health policies through a health equity lens: A measures development study protocol
Source: Front Public Health. 2022 Nov 30;10:984130. doi: 10.3389/fpubh.2022.984130 (PMC9747935; doi:10.3389/fpubh.2022.984130)
Supplement: Supplementary file 2 [file Table_2.DOCX]

Table 1: Overview of frameworks, constructs, definitions, and potential example items for surveys and interviews

| **Framework** | **Domain** | **Construct** | **Definition** | **Example Item (S = survey, I = Interview)** |
| --- | --- | --- | --- | --- |
| CFIR Damschroder et al., 2009 | Determinant | **Characteristics of Policy Implementation Leaders** | The individual(s) who lead(s) or champion(s) efforts to implement the policy (e.g., leaders among food service staff, school administrators) | How well represented are diverse racial, ethnic, gender, or identities among the group leading the policy implementation? (S) |
| Health Equity Measurement Dover & Belon, 2019 | Determinant | **Socioeconomic, Cultural and Political Context Related to Schools** | The structure of the society and the socioeconomic, political, cultural, and functional mechanisms through which it operates. Includes government apparatus, political traditions, financial institutions, transnational corporations, labor markets, citizens’ legal rights and obligations, and sociocultural values and norms, etc. | How do local school funding mechanisms (e.g., tax laws) impact policy implementation at your school? (S, I) |
| CFIR Damschroder et al., 2009 | Determinant | **Unanticipated Events** | Large-scale unanticipated events (e.g., pandemic, flood, largess in funding) | Has your school had to make changes to meal programs due to the COVID-19 pandemic? If so, what changes? (S) |
| Getting to Equity Kumanyika 2017 & 2019 | Process | **Increasing Access to Healthy Options through Policy** | Approaches that, if appropriately designed and implemented, can improve access to options for healthy eating and physical activity in socially disadvantaged communities; interventions that are core to many obesity prevention recommendations for environmental and policy change generally, and are particularly important from an equity perspective | To what extent do you think this policy benefits the most disadvantaged students in your school? (S) |
| R4P Framework Hogan 2018 | Process | **Remediate Risks** | Identify needs and exposures that put historically marginalized populations at risk for inequities, put protections in place until the harm/exposure can be structurally removed | How can nutrition inequities experienced by historically marginalized groups be mitigated through school policy implementation? (I) |
| R4P Framework Hogan 2018 | Process | **Remove Structures of Disenfranchisement** | Identify and remove structures, attitudes, beliefs, practices or experiences specific to race/ethnicity, low SES, gender, or other identities that confer disadvantage to certain groups or populations (e.g., racism, classism) | How does classism play a role in the school meal policy? (I) |
| IOF Proctor et al. 2011 | Outcome | **Appropriateness of the School Policy** | Perceived fit, relevance, or compatibility of the school meal policy for a given school setting, school staff, or students & families; and/or perceived fit of the policy to address a particular issue or problem related to health equity | How appropriate do you think the universal school meal policy is for addressing food insecurity in your school? (S) |
| CFIR Damschroder et al., 2009; IOF Proctor et al. 2011 | Outcome | **Cost of Implementing the Policy** | The financial cost of operating/implementing a policy, including resources, staff, and services needed to deliver school meals or other policy components as mandated, | To what extent do lower-resource schools in your district bear a disproportionate cost burden to successfully implement the policy? (S) |
| IOF Proctor et al. 2011 | Outcome | **Feasibility of the School Policy** | The extent to which a school meal policy can be successfully used or carried out within a school setting to advance health equity | How easily can schools obtain culturally appropriate foods that meet the nutrition standards set forth by the policy? (S) |

Note: CFIR = Consolidated Framework for Implementation Research; IOF = Implementation Outcomes Framework; R4P = Remove, Repair, Restructure, Remediate and Provide; Original framework constructs and definitions adapted in some cases to specify for the school policy context. Example items for clarification purposes only and may not accurately reflect final survey or interview questions. Response scales for fixed response survey items not included in the table.
